# Geospatial Settlement Data and Immunization Reach in Polio SIAs: Evidence from Axis of Intractable Transmission States in Northern Nigeria

**DOI:** 10.12688/gatesopenres.16387.1

**Published:** 2026-06-02

**Authors:** Abubakar Shehu, Loveth Metiboba, Nnenna Ohiaeri, Oluwatosin Ilesanmi, David Akpan, Busayo Fashoto, Glory Ugochukwu Nwakaogor, Atef Fawaz, Ota Akhigbe, Adedayo Odukoya, Oluwadamilare Akindipe, Friday Daniel

**Affiliations:** 1 eHealth Africa, Maitama, FCT, 900271, Nigeria

**Keywords:** cVDPV2, polio, Master List of Settlements, settlement tracking, polio SIAs, Vaccination, GTS.

## Abstract

**Background:**

The persistent transmission of circulating vaccine-derived poliovirus type 2 (cVDPV2) in Northern Nigeria, especially in the Axis of Intractable Transmission (AIT) states, Kebbi, Sokoto, Katsina, and Zamfara, despite national certification of wild poliovirus interruption, is strongly associated with fragmented settlement visibility, population denominator inaccuracies, and uneven implementation of Supplementary Immunization Activities (SIAs). This study examines whether these geospatial investments as broader ecosystem of interventions improve immunization reach and operational quality during cVDPV2 SIAs.

**Methods:**

A retrospective operational analysis was conducted using data from nine consecutive SIAs (April 2024–June 2025). We extracted state-level indicators from the Master List of Settlements (MLoS) geospatial records and campaign data, which included (1) geospatial completeness, defined as the proportion of settlements with validated coordinates; (2) settlement tracking coverage; (3) MLoS-derived population targets for children; and (4) number of children vaccinated. Descriptive statistics, bivariate exploratory relationships, and temporal learning analysis were performed to evaluate how geospatial status influenced tracking outcomes.

**Results:**

Geospatial completeness ranged from 92.1% (Zamfara) to 100% (Kebbi) and was associated with higher, more stable tracking coverage. Kebbi, with complete geolocation of settlements, demonstrated consistently strong tracking performance (mean 93%). Settlement tracking correlated positively with vaccination outputs in Kebbi and Sokoto but demonstrated saturation effects in Katsina and diminishing returns in Zamfara. Operational learning was observed in Sokoto, Katsina, and Zamfara, each demonstrating 8 to 10 percentage point improvements in tracking coverage across rounds.

**Conclusion:**

In addition to other contributory factors, settlement-level geolocation improves the quality and reach of cVDPV2 SIAs by strengthening visibility of target populations and promoting more reliable tracking. The MLoS updates and tracking of settlements and vaccination teams demonstrated transformative potential in optimizing vaccination campaigns by improving equity, accountability, and efficiency. Its deployment offers valuable lessons for enhancing immunization efforts in resource-constrained and security-compromised settings, emphasizing the importance of strategic integration and sustained investments in public health innovation.

## Introduction

The resolution of the 41st World Health Assembly in 1988 for the worldwide eradication of polio marked the launch of the Global Polio Eradication Initiative (GPEI), spearheaded by national governments, the World Health Organization (WHO), Rotary International, the Centers for Disease Control and Prevention, and the United Nations Children’s Fund and supported by key partners. ^1^ The GPEI was based on 4 strategies: high coverage with oral polio vaccine in routine immunization, surveillance for acute flaccid paralysis, supplemental immunization activities (SIAs), and mop-up immunizations. The consistent application of the 4 strategies by most countries subsequently led to the global declaration of eradication of wild-type polioviruses (WPVs) 2 and 3 in 2015 and 2019, respectively, ^2^ and the World Health Organization (WHO) African region was declared WPV2 free in 2020. ^3^ In addition, the emergence of circulating vaccine-derived polioviruses (cVDPVs) has led to increasing case reports of acute flaccid paralysis (AFP), which is the primary sign of polio infection, throughout the globe. Over 3000 polio cases have been reported worldwide since 2020, including nearly 700 cases in the 12 months before March 2023. ^4^ Although polio case counts are highest in low-income countries affected by war or geographic barriers to immunization programs, polio has also been detected in a growing number of middle- and high-income nations. ^5^

### Global polio eradication situation in Nigeria

Nigeria, the seventh most populous country in the world, was declared free of WPV in 2020 but remains among the largest sources of circulating vaccine-derived poliovirus type 2 (cVDPV2) globally. ^6^ Much of the risk for cVDPV outbreaks can be linked to a combination of inaccessibility, insecurity, a high concentration of zero-dose and under-immunized children, and population displacement. ^7^

Vaccine delivery in Nigeria is managed by the National Program on Immunization (NPI), which uses vaccines approved by the WHO. ^8^ Six vaccines have been used to stop polio transmission and eliminate wild poliovirus (WPV) in Nigeria. ^9^ Until 2016, the trivalent oral polio vaccine (tOPV), containing Sabin strains of all three poliovirus serotypes, was in use. ^10^ In 2015, an inactivated polio vaccine (IPV) dose was added at 14 weeks to the routine schedule. In April 2016, tOPV was replaced by the bivalent oral polio vaccine (bOPV), which included only Sabin serotypes 1 and 3. Monovalent oral polio vaccines for types 1, 2, and 3 were also introduced to target each serotype specifically. ^11^ Nigeria continued to face cVDPV2 outbreaks even after administering mOPV2 from 2018 to 2022. ^9^ In 2021, a second IPV dose was added at six weeks of age, and a new oral poliovirus vaccine, type 2 (nOPV2), was introduced. ^12^

In 2023, the National Primary Health Care Development Agency (NPHCDA), in partnership with the Global Polio Eradication Initiative (GPEI) and other stakeholders, prioritized eliminating all forms of poliovirus transmission worldwide. ^13^ The northern part of Nigeria was identified as a critical region, focusing on specific local government areas (LGAs) with the highest number of unimmunized children. ^14^ To support the polio response and stop the spread of cVDPV2, eHealth Africa received grants from the Gates Foundation in 2024 to implement the Geospatial Tracking System project.

This retrospective analysis assessed whether geospatial investments contributed to the improvement of immunization coverage and operational quality during Supplementary Immunization Activities (SIAs) from April 2024 to June 2025 across four axes of Intractable Transmission States (AITs) in northern Nigeria: Kebbi, Sokoto, Katsina, and Zamfara. Overall, this analysis highlights the importance of moving from hand-drawn maps to georeferenced ones, and it also incorporates geospatial, tracking, and immunization indicators. An important limitation is that the analysis relies on aggregated state-level data and cannot assess intra-state variations at the LGA or ward level.

Nigeria was certified WPV-free in 2020, but remains one of the largest contributors to cVDPV cases globally. ^15^ Between 2018 and 2024, 53 countries reported cVDPV outbreaks, including over 20 African nations in 2024 alone. ^16^ This resurgence highlights that eradication hinges not only on vaccine availability but also on equitable, sustained, and effective immunization, especially in hard-to-reach and underserved communities. ^17^ Achieving and sustaining global polio eradication is crucial for global health security, preventing cross-border transmission, and safeguarding all populations from this debilitating disease. The post-certification period presents unique challenges as countries experience programmatic complacency and reduced political attention to polio immunization. ^16, 18^ Nigeria has made significant efforts but remains endemic for wild poliovirus (WPV) as a result of several factors, mainly because of poor quality and coverage of the SIAs. ^19^ Another hindrance was the inability to identify areas that were missed by timely intervention with vaccination to provide the required herd immunity to break the polio transmission. ^20^

### Project context

To support the polio response, eHealth Africa received grants from the Gates Foundation in 2024 to implement the Geospatial Tracking System project, intended to strengthen operational efforts to interrupt and ultimately stop the spread of cVDPV2. The project aimed to improve geographical coverage of settlements and reduce the number of missed areas; increase access to and use of GTS data through an up-to-date geodatabase to support government and partner decision-making; reduce missed children; strengthen vaccination team performance and accountability; and improve reach to chronically missed settlements while continuously updating the Polio Master List of Settlements for northern states. Core activities included tracking vaccination teams with the GTS across 120 LGAs, supporting 19 states and the FCT in updating the Master List of Settlements (MLOS), supporting the development of under-five line-lists across seven states, and conducting capacity-building sessions in 19 states and the FCT. Powered by Novel-T, the GTS provided organizations with visibility into field activities by enabling geospatial tracking of field teams, remote retrieval of movement data, and robust analysis of tracking outputs to inform operational decisions. This assessment aimed to determine the geospatial completeness of settlement mapping and the extent to which the completeness of the geospatial Master List of Settlements datasets improves coverage rates of polio immunization campaigns in the axis of intractable transmission in Northern Nigeria.

### Methodology

This retrospective analysis utilized programmatic data collected during Supplementary Immunization Activities (SIAs) conducted between April 2024 and June 2025 across four Axis of Intractable Transmission States (AITs) in Northern Nigeria which are Kebbi, Sokoto, Katsina, and Zamfara states. The analysis focused on settlement-level geolocation quality, operational indicators, and vaccination outcomes.

The campaign datasets generated during this timeframe were analyzed to evaluate the geolocation quality based on the completeness, accuracy, consistency of georeferenced settlement data and determine the effectiveness of settlement-level tracking and explore the extent to which the geospatial completeness of the Master List of Settlements (MLoS) influences immunization coverage performance. Vaccination outcomes were derived from administrative SIA coverage data. Operational indicators included measures of settlement reach, team performance, and microplanning completeness. Between April 2024 and June 2025, a total of nine SIAs were conducted across the AIT states of Kebbi, Sokoto, Katsina, and Zamfara. These campaigns followed an intensive outbreak-response schedule, with rounds implemented approximately every 4-8 weeks. The majority were sub-national immunization days (SNIDs), complemented by periodic national immunization days (NIDs) and interspersed in-between round activities (IBRAs) to reach missed and underserved populations.

Analytical approaches included stratified comparisons across levels of geolocation quality. While stratification was used to reduce heterogeneity across settings, the analysis did not fully account for all potential confounders.

### Data sources

This study sourced data from the project monitoring and evaluation performance tracking database, including:

1. Master List of Settlements: This is the geo-coded database managed by eHealth Africa of recognized settlements in each state. The dataset has the counts of settlements with validated GIS coordinates for each campaign, also expressed as geospatial coverage percentage.
2. Settlements tracked: The database has state-level campaign data capturing the number of settlements tracked during implementation rounds and the geographical coverage, such as tracking coverage rate.
3. This study also used the vaccination records. This implies the targeted children for immunization, as determined by the MLoS estimates and the number of children immunized as reported by the campaign teams.
4. These datasets were triangulated, yielding 36 unique observations without identifiers.

### Measures

• Settlement Geospatial Completeness: Geospatial completeness was defined as the percentage of MLoS settlements with validated GPS coordinates during each campaign. This variable was interpreted as the degree of geospatial preparedness and microplanning visibility.
• Vaccination Tracking Coverage Rate: These are the campaign tracking indicators, such as:
  ○ Settlements tracked during each campaign round; and
  ○ Tracking coverage rate, representing the proportion of total mapped settlements that were reached or monitored. Tracking coverage was interpreted as the primary operational quality metric.
• **Vaccination coverage:** Vaccination outputs were assessed using:
  ○ The absolute number of children vaccinated, and
  ○ An immunization ratio, defined as children vaccinated divided by children targeted for that campaign.

### Data analysis

**Descriptive analyses**

In this study, we applied the state-level means and distributions to summarize geospatial completeness, settlement tracking, and vaccination performance across campaign rounds. Temporal trends were inspected to identify improvements in operational performance.

**Association between MLoS completeness and tracking performance**

To assess whether settlement geospatial completeness was associated with tracking coverage, mean geospatial coverage and mean tracking coverage were compared across states. Given the limited number of states (n = 4), emphasis was placed on directionality and consistency rather than statistical inference. The analysis evaluated whether more complete geospatial readiness translated into more stable tracking and operational performance.

**Relationship between settlement tracking and vaccination outputs**

Pearson correlation coefficients were computed between the number of settlements tracked and the number of children vaccinated, both across the pooled dataset and within individual states. Subnational stratification was required because terrain, population mobility, service access, and risk patterns differ across states, potentially influencing the relationship between geographic reach and population yield.

**Temporal analysis of operational learning**

To assess performance change over time, campaign rounds were indexed chronologically within each state. Tracking coverage was assessed across early rounds (1–3) and late rounds (7–9). Improvements were interpreted as evidence of operational adaptation, including settlement list refinement and improved supervisory routines.

**Data treatment and quality assurance**

The missing data were handled through within-state forward fill for categorical descriptors to maintain longitudinal continuity. No imputation was performed for population or immunization variables to avoid inflating performance metrics. The state-level differences were preserved. There was no pooling or normalization that was performed beyond descriptive averages, because heterogeneity was intrinsic to the research question.

### Statistical interpretation and analytical considerations

The analytical approach used in this study quantifies the association between settlement-level geolocation quality and vaccination outcomes during Supplementary Immunization Activities (SIAs). The model is designed to estimate the strength and direction of this relationship while accounting for selected observable factors through stratification and/or adjustment. However, the model does not establish causality. The findings should therefore be interpreted as evidence of correlation rather than proof that improvements in geolocation quality directly led to improved vaccination performance. The observed association may reflect underlying contextual conditions that simultaneously influence both variables.

## Results

### Geospatial completeness of settlement mapping

Across the study period, the completeness of geospatial coordinates in the Master List of Settlements (MLoS) was consistently high in all four high-risk northern states ( Table 1). Kebbi state demonstrated full geocoordinate completeness, achieving almost 100% mapping coverage across all campaign rounds. Katsina and Sokoto states followed closely, with mean completeness of 97.3% and 96.7% respectively, while Zamfara had the lowest level of mapping maturity at 92.1%.

**Table 1.** Settlements geocoded across Kebbi, Katsina, Sokoto, and Zamfara states.

States	MLoS with coordinates	% of Settlements with coordinates
Kebbi	18,190	99.77%
Katsina	24,843	97.30%
Sokoto	10,525	96.70%
Zamfara	10,838	92.10%

Source: GTS study geodata

Legend: Shows the Master List of Settlements with coordinates.

Abbreviations: MLOS meaning Master List of Settlements.

Source: The evaluation study.

### Results of settlement tracking

**Figure 1.**
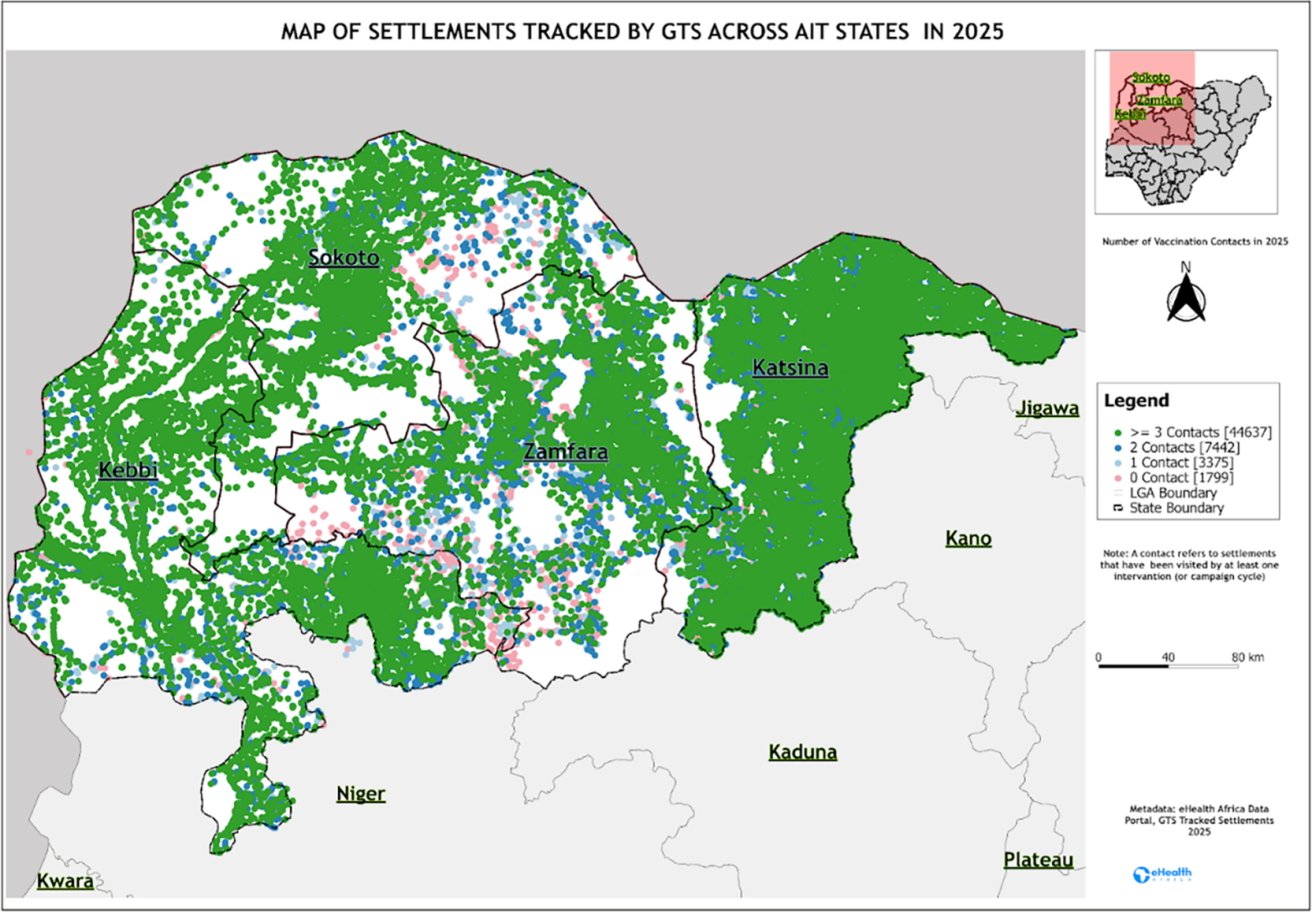
Map of AITS, including the settlement tracked. Legend: The figure shows the countries where GTS was implemented during campaign rounds.

The analysis of the tracking data shows variation across campaign rounds. In Sokoto state, settlements tracked increased from 7,789 in March 2024, when the intervention began, to 10,572 in October 2025, with a mean of 7,982 settlements tracked per campaign. Katsina consistently had the highest settlement tracked, with an average of 12,870 settlements per round. In Kebbi, the settlements tracked were above 13,000 in most rounds, with an average of 9,987 settlements tracked per campaign. Zamfara recorded the lowest tracking data, with an average of 7,072 settlements tracked per campaign ( Table 2, Figure 1).

**Table 2.** Records of settlements tracked per round.

	Number of settlements tracked			
Campaign rounds	Sokoto	Katsina	Zamfara	Kebbi
March 2024	7,789	9,485	6,611	7,714
April 2024	6,531	4,962	4,275	44,06
Sept 2024	11,043	19,312	10,582	13,617
Oct 2024	4,646	8,842	3,572	7,394
Nov 2024	11,622	19,411	10,090	13,836
April 2025	7,654	15,679	8,523	13,519
June 2025	9,976	18,305	9,683	15,192
Sept 2025	2,004	1,378	1,199	1683
Oct 2025	10,572	18,459	9,113	12518
Total	71,837	115,833	63,648	89,879

Source: GTS study geodata.

Legend: Number of settlements track by campaign rounds in the four states.

**Figure 2.**
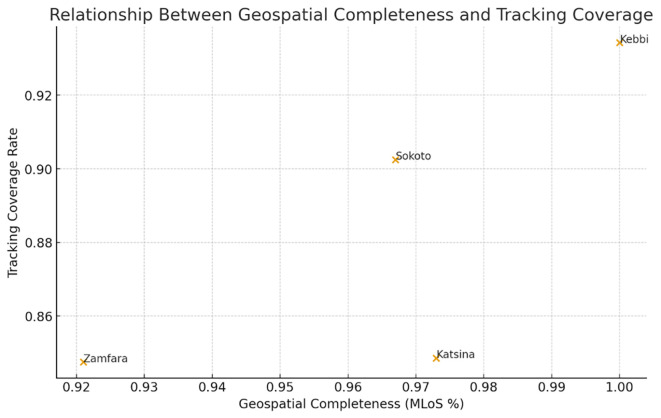
The relationship between geospatial completeness and vaccination tracking performance, using the average values across the four states. Legend: Geospatial completeness and vaccination tracking performance, using the average values across the four states.

The bivariate exploratory analysis shows that the relationship between geospatial completeness and vaccination tracking performance was evident: states with more fully geolocated settlements had higher tracking coverage during campaign delivery. Kebbi, with full geospatial mapping, maintained a mean tracking coverage of 93%, whereas Zamfara, with the lowest mapping completeness, achieved only 84.8% ( Figure 2). This pattern shows that the expansion of geospatial enumeration capabilities is directly associated with the reliability of settlement-level campaign operations. In practical terms, the presence of validated coordinates appears to reduce settlement invisibility, improve microplanning accuracy, and support stronger supervisory reach during implementation.

### Relationship between settlement tracking and immunization results

**Figure 3.**
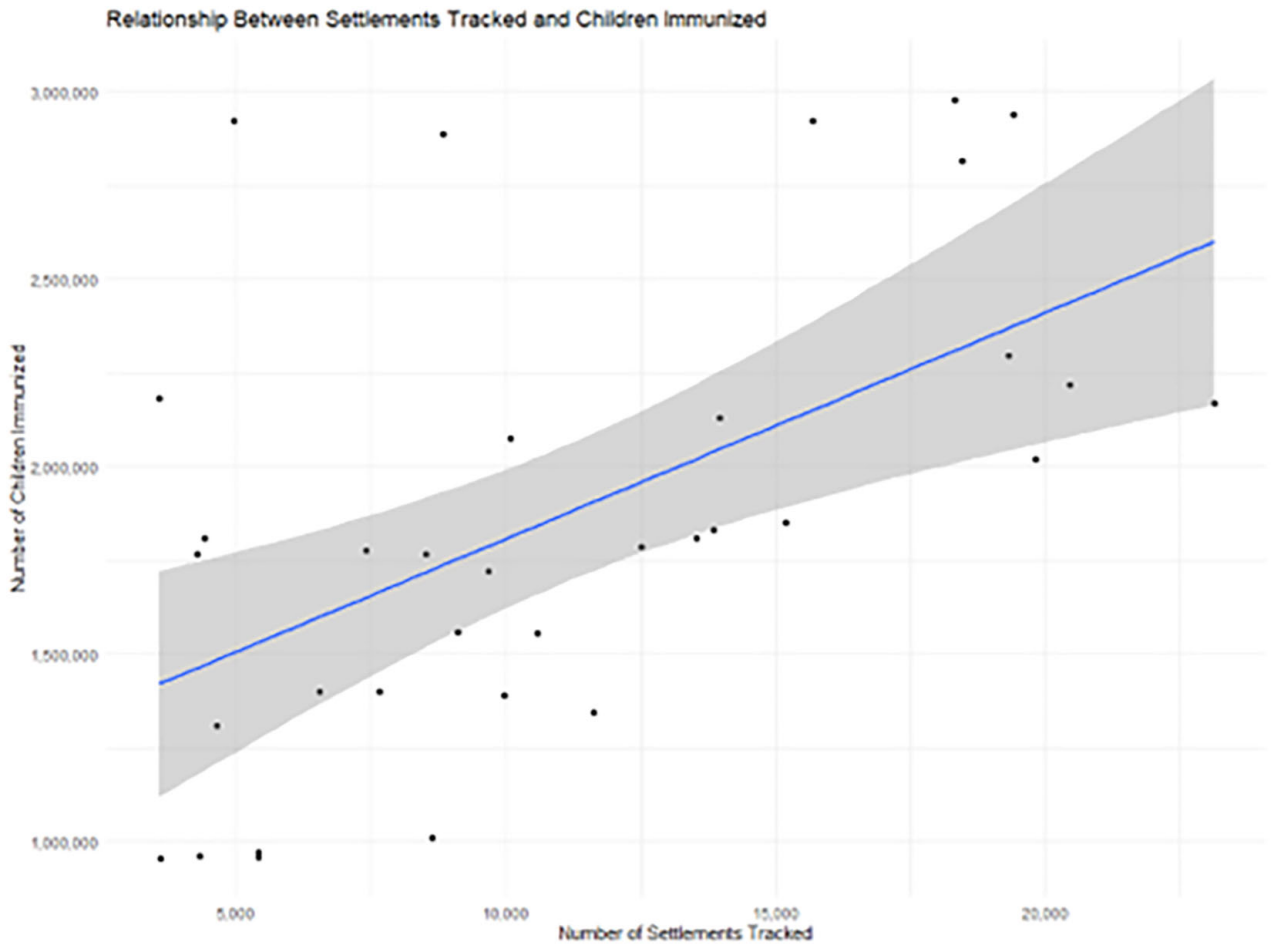
A positive correlation of 0.56 shows that tracking more settlements leads to improvement in immunization coverage. Legend: Positive correlation of tracked settlements and their immunization coverage.

The relationship between the number of settlements tracked during campaigns and the number of children vaccinated varied across states and reflected the campaign operations and their delivery. When all campaign rounds were pooled, settlement tracking was moderately positively associated with immunization results. Campaigns covering more settlements tended to vaccinate more children, indicating that geographic expansion of field activities was generally linked to wider children population reach. The correlation between settlements tracked and children immunized was 0.5560589 (approximately 0.56) ( Figure 3). This relationship was strongest in Kebbi and Sokoto, where higher tracking results had a relationship with increased immunization numbers.

In contrast, Katsina displayed a near-zero association between tracking and vaccinations ( Table 3). The number of children vaccinated remained high regardless of how many settlements were tracked, suggesting that demand, campaign saturation, or historical experience may enable the system to reach children, in addition to vaccination tracking. Zamfara demonstrated a negative association between these indicators, as campaigns that expanded settlement coverage tended to vaccinate fewer children, reflecting a dilution effect, where widening the campaign footprint resulted in lower yield per settlement, or where smaller or more remote settlements were added late in the process.

**Table 3.** Tracking coverage and immunization coverage.

State	Total targeted	Total immunized	Mean tracking coverage	Mean settlement tracked	Immunization coverage	Correlation coefficient
Kebbi	6,849,587	10,860,184	0.9233333333	11,144.166667	1.585523916	0.541
Katsina	9,823,200	17,462,140	0.8583333333	14,276.333333	1.777642723	0.025
Sokoto	9,290,511	8,395,489	0.9	8,500.166667	0.9036627802	0.433
Zamfara	6,301,625	11,073,017	0.8316666667	7542.666667	1.757168508	-0.383

Source: GTS Study geodata.

Legend: Showing correlation coefficient of total targets and total immunized in the four states.

### Operational learning across campaign rounds

**Figure 4.**
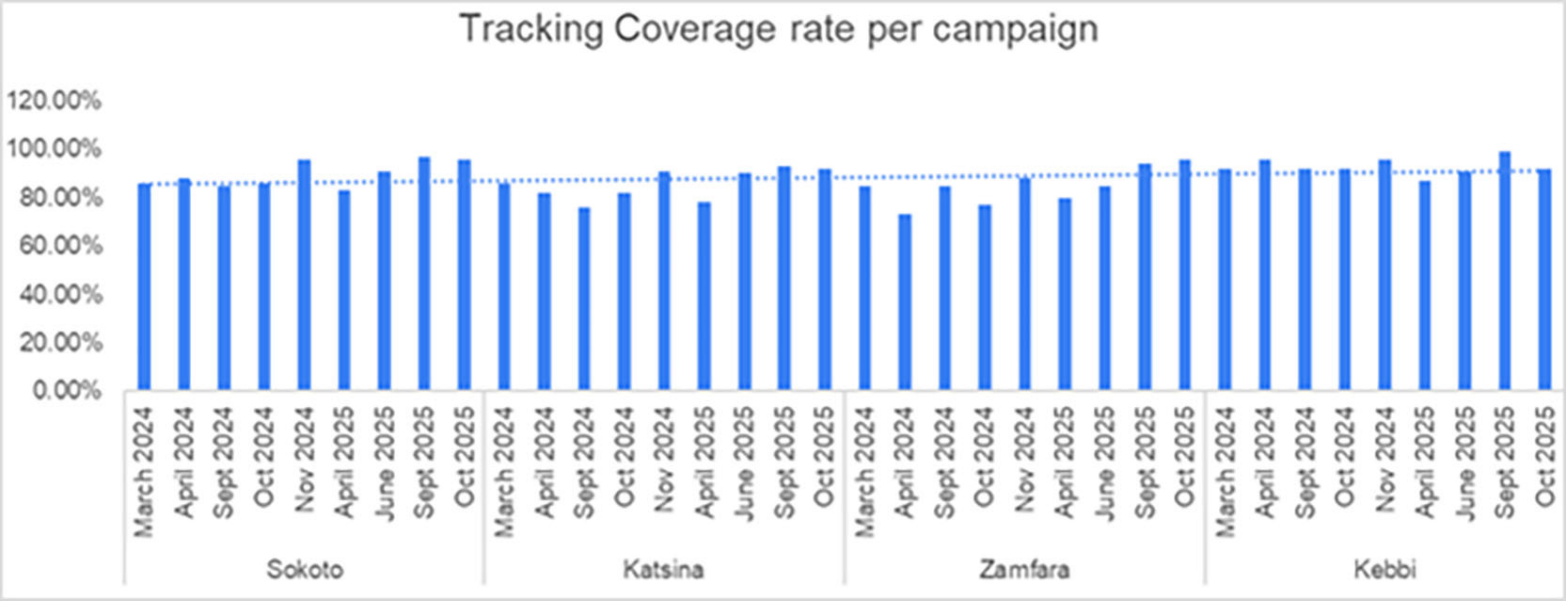
Trends in vaccination coverage across campaign rounds 2024-2025. Legend: Shows trends in vaccination coverage across campaign rounds 2024-2025 in the four states.

Tracking coverage showed evidence of operational improvement across repeated campaign cycles. When campaigns were sequenced chronologically from the first to the ninth round in each state, coverage tracking rose consistently over time in Sokoto, Katsina, and Zamfara. Sokoto improved from an average of 86.3% in its first three rounds to 94.7% in its most recent three, reflecting an 8.4 percentage point increase. Katsina and Zamfara demonstrated similar upward trajectories, each recording improvements of approximately 10 percentage points between early and late rounds. This progressive enhancement indicates campaign-level learning, likely driven by improved settlement lists, supervisory discipline, and frontline team familiarity with geospatial tools. Kebbi displayed minimal temporal change, though for positive reasons. The state began with performance at saturation levels, averaging 93.3% tracking coverage in its early rounds and maintaining 94% in later rounds ( Figure 4).

## Discussions

It is imperative to note that while this study demonstrates meaningful associations between geospatial completeness, settlement tracking, and immunization reach, these findings were interpreted with appropriate caution. Polio transmission dynamics in Northern Nigeria are shaped by a complex interplay of factors, including population mobility, insecurity, health system capacity, vaccine hesitancy, and sociocultural influences. ^21^ As such, geospatial tools, while valuable, represent only one component within a broader ecosystem of interventions required to achieve and sustain high immunization coverage.

The observed improvements in tracking performance and operational visibility associated with geospatial completeness are consistent with prior evidence from GIS-supported immunization efforts in Nigeria. However, these tools should not be interpreted as a panacea for addressing persistent transmission. Rather, they function as critical enablers that enhance microplanning precision, accountability, and supervision, particularly when integrated with strong community engagement strategies, reliable population data systems, and adaptive campaign implementation approaches.

Importantly, the heterogeneity observed across states, such as saturation effects in Katsina and diminishing returns in Zamfara, further underscores that geospatial interventions operate within context-specific constraints. Low coverage previously recorded in states such as Kebbi, Sokoto, Katsina, and Zamfara is not solely a function of technical or microplanning limitations, but also reflects deeply rooted structural and contextual challenges. ^21^ These findings reinforce the need for tailored, multi-pronged strategies that combine geospatial innovations with broader health system strengthening efforts.

This Polio vaccination campaign analysis from four high-risk northern Nigerian states shows that settlement coordinate coverage, settlement tracking performance, and the targeting of children to be vaccinated based on the MLoS updates are significant determinants of polio vaccination outcomes in high-risk settings in Nigeria. These results show the empirical value of GIS-enabled vaccination interventions in Nigeria’s polio programme, while also highlighting persistent weaknesses in population estimates.

The high levels of geospatial completeness observed in Kebbi, Sokoto, and Katsina, and to a lesser extent in Zamfara, are consistent with over a decade of investment in GIS-based vaccination interventions and team tracking in northern Nigeria. Earlier work by Barau et al. and Gammino et al. showed that the transition from hand-drawn maps to georeferenced settlement lists and GPS-tracked teams improved identification of missed settlements and reduced the likelihood of entire communities being overlooked during SIAs. ^22^^,^^23^ Our results show that states with more complete MLoS coverage tend to achieve higher and more stable tracking coverage, with Kebbi, where geospatial completeness reached 100%, maintaining the highest average tracking coverage across campaigns. This aligns with prior evaluations from Bauchi and Sokoto, which found that GIS-supported campaigns increased the proportion of planned settlements actually visited and improved the outcomes of routine immunization sessions. ^22– 24^ Conceptually, geospatial completeness acts as a campaign visibility layer.

These findings add to the global evidence showing that GIS-enabled campaigns are not just a technological innovation but a structural determinant of equity in vaccination campaigns. Gavi’s synthesis of GIS use in immunization programming notes that geospatial approaches have been particularly effective in hard-to-reach, insecure, and migratory settings, including northern Nigeria. ^25^

The association between settlement tracking and immunization output varied meaningfully by state. In Kebbi and Sokoto, campaigns that tracked more settlements tended to vaccinate more children, indicating that geographic expansion was accompanied by proportional gains in coverage. These patterns are consistent with earlier GIS-tracking pilots, which showed that systematically monitored teams were more likely to complete their planned routes, reach all assigned settlements, and identify previously missed locations. ^19^

In Katsina, immunization coverage remained high even when the number of tracked settlements fluctuated, suggesting a level of saturation or other determining factors. In Zamfara, however, expanding the settlement tracking was associated with lower vaccination yield per settlement, possibly because marginal settlements were smaller, more remote, or faced security challenges. This is consistent with evidence from mass campaign literature showing that high-frequency or wide-footprint campaigns can strain local health systems and sometimes generate diminishing returns in hard-to-reach or conflict-affected areas. ^26^

One of the most critical findings of this assessment is the frequent occurrence of immunization ratios above 1.0, often substantially so, particularly in Katsina. This phenomenon of vaccinating more children than targeted from the MLoS has been described in administrative coverage literature and is typically attributed to inaccuracies in the denominator (targeted population) rather than exceptional programme performance. ^26^

In low- and middle-income countries, denominators for coverage calculations are commonly derived from census projections, outdated population registers, or local estimates by health workers, all of which may fail to account for rapid demographic change, urbanisation, migration, and security events. ^27^ Importantly, we observed that higher tracking coverage was associated with immunization ratios closer to 1.0, suggesting that robust settlement tracking can help stabilize denominators/targeted children population. This finding aligns with the work from Kenya and broader WHO/UNICEF technical guidance, which emphasizes that improving the quality of target population estimates, through integration of geospatial data, survey findings, and updated demographic models, is critical to producing credible coverage estimates. ^28^ In the Nigerian context, initiatives that reconcile MLoS data, GRID3 population grids, and DHIS2 reporting could help correct systemic underestimation in polio and other vaccine campaigns. ^29^

The distinct operational results emerging from this analysis can be used programmatically to inform differentiated support:

• Geospatially mature states such as Kebbi can serve as learning hubs, contributing best practices in MLoS maintenance, supervisory dashboards, and the use of GIS in microplanning.
• Balanced states like Sokoto illustrate how geospatial readiness and realistic population estimates can align to produce credible coverage data, similar to earlier GIS microplanning successes documented there.
• High-throughput states such as Katsina need targeted work on denominator realism, through integration of geospatial population estimates, zero-dose mapping, and survey triangulation, rather than on sheer campaign frequency.
• Structurally fragile states like Zamfara require foundational strengthening of settlement mapping, security-sensitive outreach strategies, and explicit trade-off analysis between breadth and depth of coverage.

These differentiated strategies are aligned with national polio emergency plans and post-certification transition frameworks, which call for focused use of geospatial tools and campaign assets to support routine immunization and other vaccine-preventable disease control efforts. ^30^

## Conclusion

This study provides evidence that improved settlement-level geolocation quality is positively associated with better vaccination performance during Supplementary Immunization Activities (SIAs) in Northern Nigeria. These findings reinforce the value of geospatial tools in strengthening microplanning, improving settlement identification, and supporting more systematic campaign implementation. However, the results should be interpreted with appropriate caution. The analysis does not establish a causal relationship between geolocation quality and vaccination outcomes, and the observed associations are likely influenced by underlying contextual factors such as geographic accessibility, security conditions, health system capacity, and community engagement. Settlements that are easier to reach, both physically and socially, may inherently exhibit both higher geolocation accuracy and better vaccination coverage, suggesting that geospatial improvements operate within, rather than independently of, these broader enabling conditions.

Importantly, the study highlights those persistent gaps in SIA performance across states such as Kebbi, Sokoto, Katsina, and Zamfara cannot be fully explained by technical or operational factors alone. Social dynamics, including community trust, competing health priorities, and local political and security realities, play a critical role in shaping immunization outcomes. As such, geospatial approaches should be viewed as complementary tools that enhance planning and accountability but are not sufficient in isolation to address systemic challenges.

Taken together, these findings suggest that maximizing the impact of geospatial interventions requires their integration into a broader, context-sensitive strategy that simultaneously addresses access constraints, strengthens frontline capacity, and builds community trust. Future research should focus on longitudinal and mixed method approaches to better disentangle the pathways through which geospatial tools contribute to immunization performance and to identify the conditions under which their benefits are most pronounced.

### Strengths and limitations

This study has several strengths. It leverages real-world operational data across multiple SIA rounds and states, allowing examination of temporal trends and cross-state heterogeneity. It also integrates geospatial, tracking, and immunization indicators, highlighting relational dynamics rather than focusing on a single metric such as administrative coverage. The findings are grounded in a robust body of evidence on GIS-supported microplanning and target population estimation challenges, and they speak directly to programme decision-makers tasked with sustaining high-quality campaigns in the post-certification era. However, this study has several limitations that should be considered when interpreting the findings. First, the analysis is based on observational data, which limits the ability to establish causal relationships between settlement-level geolocation quality and vaccination outcomes. While the study demonstrates an association between improved geolocation and higher vaccination performance, this relationship should be interpreted with caution, as it may be influenced by unmeasured confounding factors.

Geographic accessibility, security conditions, and social acceptance of immunization activities are likely to simultaneously influence both the quality of geolocation data and vaccination coverage. Settlements that are easier to access, both physically and socially, are more likely to have accurate geospatial data and also to achieve higher vaccination coverage. Although the analysis attempts to control for some of these factors through stratification, residual confounding is likely, and the observed associations may therefore overestimate the independent effect of geolocation quality.

Second, the study may be affected by measurement limitations related to both geolocation quality and vaccination outcomes. Geolocation accuracy may vary depending on the tools used, the skill level of field teams, and environmental factors such as terrain or network connectivity. Similarly, vaccination coverage estimates may be subject to reporting bias, including over-reporting or under-reporting during Supplementary Immunization Activities (SIAs), especially in settings where administrative data systems are relied upon.

Third, the analysis does not fully capture important contextual and social determinants that influence immunization performance. Factors such as community trust, political dynamics, caregiver perceptions, competing health priorities, and local leadership engagement play a critical role in vaccination uptake but are not directly measured in the dataset. In regions where communities may express frustration with the prioritization of polio over other high-burden diseases such as malaria, these dynamics can significantly affect campaign performance independent of geospatial data quality.

Fourth, security constraints and population mobility in the study areas, particularly in states such as Katsina, Sokoto, Zamfara, and Kebbi, may affect both data quality and program implementation. Insecurity can restrict access to certain settlements, resulting in incomplete geolocation data and missed vaccination opportunities. Additionally, migratory and nomadic populations may not be fully captured in static geospatial datasets, leading to potential underestimation of target populations and gaps in coverage.

Fifth, heterogeneity across states and local government areas is not fully explored in the analysis. Differences in infrastructure, terrain, governance, health system capacity, and implementation practices may influence both geolocation and vaccination outcomes in ways that are not adequately accounted for. As a result, findings from one context may not be directly generalizable to others.

Sixth, the study focuses primarily on technical and operational aspects of geospatial implementation and may underrepresent broader programmatic factors. Issues such as workforce capacity, training quality, supervision, and resource availability can significantly influence both data quality and vaccination performance but are not explicitly examined in the analysis.

Seventh, the temporal scope of the data may limit the ability to assess longer-term impacts of geospatial interventions. The study captures outcomes within a specific campaign period and may not reflect sustained improvements or changes over time. Longitudinal analyses would be required to better understand the durability of observed effects.

Finally, while the study contributes to the growing evidence base on geospatial tools in immunization programs, it should be viewed as part of a broader body of work rather than as definitive evidence of impact. Geospatial interventions operate within complex health systems, and their effectiveness depends on how they are integrated with other strategies addressing social, political, and operational challenges. Future research and programme priorities.

Future work should extend this analysis in three directions. First, linking settlement-level geospatial data, GPS tracks, and independent coverage surveys would allow a better assessment of zero-dose and under-immunized populations. Second, linking GPS data tracks with walk-through population estimates to forecast resources required at the ward level. Third, identify the prevailing factors that may have impacted the increase in immunization coverage even when the number of tracked settlements fluctuated, suggesting a level of saturation.

### Future research and programme priorities

Future work should extend this analysis in three directions. First, linking settlement-level geospatial data, GPS tracks, and independent coverage surveys would allow a better assessment of zero-dose and under-immunized populations. Second, linking GPS data tracks with walk-through population estimates to forecast resources required at the ward level. Third, identify the prevailing factors that may have impacted the increase in immunization coverage even when the number of tracked settlements fluctuated, suggesting a level of saturation.

## Ethical considerations

The analysis used state-level operational data with no patient identifiers. All records originated from routine immunization monitoring systems and contained no personal information. Ethical approval was not required under national guidelines governing the analysis of secondary operational data used for program improvement.

## Generative AI tools statement

The authors declare that no Generative AI tools were used in preparing the manuscript.

## Data Availability

The original contributions and data presented in the study are included in the article material on the Open Science Framework: https://doi.org/10.17605/OSF.IO/5YCQ6. ^31^

## References

[1] World Health Organization: Poliomyelitis (Polio). Accessed December 15, 2025. Reference Source

[2] *Polio Eradication Strategy 2022–2026: Delivering on a promise.* Geneva: World Health Organization;2021. Licence: CC BY-NC-SA 3.0 IGO.

[3] World Health Organization African Region: Africa eradicates wild poliovirus. 25 Aug 2020. Accessed November 30, 2025. Reference Source

[4] Durrheim DN : Eliminating all circulating vaccine-derived poliovirus: a prerequisite to declaring global polio eradication. *Int. Health.* 2023 Mar 1;15(2):109–110. 10.1093/inthealth/ihac068 36271900 PMC9977216

[5] Global Polio Eradication Initiative: History of polio. Reference Source

[6] World Health Organization|Regional: Office for Africa Protecting Every Child in Nigeria from Poliovirus. 2023. Reference Source

[7] World Health Organization : Statement of the forty-first meeting of the Polio IHR Emergency Committee. 2025. Reference Source

[8] Oyadiran OT Usman SA Osoba ME : Towards effective and efficient COVID-19 vaccination in Nigeria. *J. Glob. Health Reports.* 2021;5:e2021023. 10.29392/001c.21404 Reference Source

[9] Eze OV Meyer JC Campbell SM : Poliomyelitis in Nigeria: Impact of Vaccination Services and Polio Intervention and Eradication Efforts. *Vaccines.* 2025;13(3):232. 10.3390/vaccines13030232 40266076 PMC11945573

[10] Bassey BE Braka F Vaz RG : The global switch from trivalent oral polio vaccine (tOPV) to bivalent oral polio vaccine (bOPV): facts, experiences and lessons learned from the south-south zone; Nigeria, April 2016. *BMC Infect. Dis*. 2018;18(1):57. 10.1186/s12879-018-2963-6 29374467 PMC5787308

[11] Tevi-Benissan C Okeibunor J Châtellier GM du : Introduction of Inactivated Poliovirus Vaccine and Trivalent Oral Polio Vaccine/Bivalent Oral Polio Vaccine Switch in the African Region. *J. Infect. Dis.* 20171;216(suppl_1):S66–S75. 10.1093/infdis/jiw616 28838178 PMC5853502

[12] Zubairu I Abba AD Visalakshi J : Impact of novel Oral Poliovirus Type 2 vaccination campaigns: A seroprevalence survey in Nigeria, 2022. *Vaccine.* 2025;54:126978.0264–410X. 10.1016/j.vaccine.2025.126978 https://www.sciencedirect.com/science/article/pii/S0264410X25002750 40112661 PMC12132045

[13] Global Polio Eradication Initiative Annual Report 2023. (Accessed 17 April 2026). https://reliefweb.int/report/world/global-polio-eradication-initiative-annual-report-2023

[14] Global Polio Eradication Initiative : A Critical Year for polio eradication efforts in northern Nigeria. 2023. https://polioeradication.org/news/2023-a-critical-year-for-polio-eradication-efforts-in-northern-nigeria/

[15] Bricks LF Macina D Vargas-Zambrano JC : Polio Epidemiology: Strategies and Challenges for Polio Eradication Post the COVID-19 Pandemic. *Vaccines (Basel).* 2024 Nov 26;12(12):1323. 10.3390/vaccines12121323 39771986 PMC11680066

[16] Sun Y Keskinocak P Steimle LN : Modeling the spread of circulating vaccine-derived poliovirus type 2 outbreaks and interventions: A case study of Nigeria. *Vaccine X.* 2024 Mar 16;18:100476. 10.1016/j.jvacx.2024.100476 38617838 PMC11011220

[17] Shattock AJ Johnson HC Sim SY : Affiliations Expand Contribution of vaccination to improved survival and health: modelling 50 years of the Expanded Programme on Immunization. *Lancet.* 2024 May 25;403(10441):2307–2316. 10.1016/S0140-6736(24)00850-X 38705159 PMC11140691

[18] Ahmad T Baig M Khan M : Polio eradication in Pakistan: Hope against hope or are we near eradication? *J. Virus Erad.* 2024 Apr 7;10(1):100371. 10.1016/j.jve.2024.100371 38618138 PMC11011219

[19] Touray K Mkanda P Tegegn SG : Tracking Vaccination Teams During Polio Campaigns in Northern Nigeria by Use of Geographic Information System Technology: 2013-2015. *J. Infect. Dis.* 2016 May 1;213 Suppl 3(Suppl 3):S67–S72. Epub 2015 Nov 25. 10.1093/infdis/jiv493 26609004 PMC4818548

[20] Atagbaza A Okeibunor J Amadou F : Vaccinations and Vaccinators’ Tracking System in Island Settlements: CHAD 2017-2018. *J. Immunol. Sci.* 2021 Apr 12;Spec Issue(2):1116. 10.29245/2578-3009/2021/S2.1116 33954308 PMC7610726

[21] Kuuyi A Kogi R : Factors contributing to immunization coverage among children less than 5 years in Nadowli-Kaleo District of Upper West Region, Ghana. *PLOS Glob. Public Health.* 2024 Aug 1;4(8):e0002881. 10.1371/journal.pgph.0002881. 39088462 PMC11293730

[22] Barau I Zubairu M Mwanza MN : Improving polio vaccination coverage in Nigeria through the use of geographic information system technology. *J. Infect. Dis.* 2014 Nov 1;210 Suppl 1:S102–S110. 10.1093/infdis/jiu010 25316823

[23] Gammino VM Nuhu A Chenoweth P : Using geographic information systems to track polio vaccination team performance: pilot project report. *J. Infect. Dis.* 2014 Nov 1;210 Suppl 1:S98–S101. 10.1093/infdis/jit285 25316882

[24] Oteri J Hussaini MI Bawa S : Application of the Geographic Information System (GIS) in immunisation service delivery; its use in the 2017/2018 measles vaccination campaign in Nigeria. *Vaccine.* 2021;39 Suppl 3(3):C29–C37. 0264-410X. 10.1016/j.vaccine.2021.01.021 33478790

[25] Chaney SC Mechael PN : Using Geospatial Technologies to improve immunisation coverage and equity. A landscape analysis and theory of change. 2020. Reference Source

[26] Michael CA Ashenafi S Ogbuanu IU : An Evaluation of Community Perspectives and Contributing Factors to Missed Children During an Oral Polio Vaccination Campaign – Katsina State, Nigeria. *J. Infect. Dis.* 210 Suppl 1(210)1:S131–S135. 10.1093/infdis/jiu288 25316827

[27] Stashko LA Gacic-Dobo M Dumolard LB : Assessing the quality and accuracy of the national immunization program reported target population estimates from 2000 to 2016. *PLoS ONE.* 2019;14:e0216933. 10.1371/journal.pone.0216933 31287824 PMC6615593

[28] Karanja-Chege C Agweyu A Were F : Addressing the challenges of estimating the target population in calculation of routine infant immunization coverage in Kenya. *PLOS Glob. Public Health.* 2025;5:e0004298. 10.1371/journal.pgph.0004298 40627639 PMC12237011

[29] Faisal-Shuaib F Garba AB Meribole E : Implementing the routine immunisation data module and dashboard of DHIS2 in Nigeria, 2014–2019. *BMJ Glob. Health.* 2020;5:e002203. 10.1136/bmjgh-2019-002203 32694218 PMC7375433

[30] Africa Field Epidemiology Network/Africa Health Budget Network (Zero-Dose Learning Hub): Closing The Immunization Gap: Enhancing Routine Immunization in Nigeria by Reaching Zero-Dose and Under Immunized Children in Marginalized Communities: Report of a Rapid Assessment. 2024. Reference Source

[31] Metiboba L Nwakaogor GU : Geospatial Settlement Data and Immunization Reach in Polio SIAs: Evidence from Axis of Intractable Transmission States in Northern Nigeria. 2025, December 15. Reference Source 10.12688/gatesopenres.16387.1 PMC13230485 42244932

